# What Drives Influenza Vaccination in People with Diabetes? Evidence from the National Health Surveys of 2020 and 2023 in Spain

**DOI:** 10.3390/epidemiologia7020053

**Published:** 2026-04-08

**Authors:** Tomás Chivato-Martín-Falquina, Jose J. Zamorano-Leon, Ana Lopez-de-Andres, Lucia Fuentes-Arroyo, Rodrigo Jimenez-Garcia

**Affiliations:** 1 Unidad de Radiofarmacia, Hospital General Universitario Santa Lucía, 30202 Cartagena, Spain; tomas.chivato@carm.es; 2 Department of Public Health and Maternal & Child Health, Faculty of Medicine, Universidad Complutense de Madrid, Instituto de Investigación Sanitaria del Hospital Clínico San Carlos (IdISSC), 28040 Madrid, Spain; luciaifu@ucm.es (L.F.-A.); rodrijim@ucm.es (R.J.-G.); 3 Department of Public Health and Maternal & Child Health, Faculty of Pharmacy, Universidad Complutense de Madrid, Instituto de Investigación Sanitaria del Hospital Clínico San Carlos (IdISSC), 28040 Madrid, Spain; anailo04@ucm.es

**Keywords:** diabetes, flu vaccine, vaccination coverage, immunization, health survey, Spain, COVID-19

## Abstract

**Background/Objectives**: Diabetes is associated with an increased risk of influenza-related complications; therefore, annual vaccination constitutes an essential preventive measure. The objective of this study is to analyze the evolution of influenza vaccination coverage among the population with diabetes in Spain between 2020 and 2023 and to identify factors associated with adherence, comparing it with a matched population without diabetes. **Methods**: A cross-sectional study was conducted using data from the European Health Survey in Spain 2020 and the Spanish National Health Survey 2023, applying 1:1 matching by age, gender, and place of residence. Multivariable logistic regression was applied to assess time trend and to identify adherence predictors. **Results**: Vaccination coverage among individuals with diabetes increased from 52.0% in 2020 to 65.9% in 2023 and was higher than that observed among the matched participants without diabetes in both periods. Older age and the presence of comorbidities, such as myocardial infarction or respiratory diseases, were associated with a higher likelihood of vaccination, whereas alcohol consumption and smoking were associated with lower adherence among subjects with diabetes. The year 2023 was independently associated with a higher probability of vaccination compared with 2020 (OR: 1.82; 95% CI: 1.56–2.12). **Conclusions**: Although influenza vaccination coverage among the Spanish people with diabetes has improved following the COVID-19 pandemic, it remains below recommended targets, highlighting the need to strengthen targeted strategies aimed at less adherent subgroups.

## 1. Introduction

Diabetes is a highly prevalent chronic disease that has a significant impact on morbidity, mortality, and healthcare systems. In patients with diabetes, infection with the influenza virus is associated with severe cardiovascular events, hospitalization, and even premature mortality [1,2,3]. For this reason, international guidelines recommend annual influenza vaccination for all individuals with diabetes, regardless of disease type or treatment regimen [4,5].

Unfortunately, influenza vaccination coverage among people with diabetes in Spain remains suboptimal, ranging in recent years from 52.6% to 53.4% according to the latest Spanish National Health Surveys from 2011 and 2017 and the European Health Surveys in Spain from 2014 and 2020 [6]. In fact, these figures are far below the 75% coverage target established by the World Health Organization and the European Centre for Disease Prevention and Control for older adults and patients with chronic diseases, including diabetes [7,8]. Among the causes of this low vaccination coverage are classical factors related to a low perception of influenza risk and fear of adverse effects [9,10]. However, sociodemographic and health-related variables also appear to be closely associated with adherence to influenza vaccination in populations with diabetes [6,10]. Additionally, with the emergence of the COVID-19 pandemic in 2020, new factors have arisen that could affect adherence to influenza vaccination in this population [11]. In this regard, the effects that the COVID-19 pandemic and the strengthening of vaccination campaigns and public awareness of COVID-19 immunization may have had on influenza vaccination coverage among the diabetes population remain unknown.

All the above underscores the importance of analyzing influenza vaccination rates in high-risk populations, such as individuals with diabetes, as well as identifying factors associated with adherence to vaccination. Therefore, the aim of this study was to analyze and compare influenza vaccination coverage among people with diabetes residing in Spain in 2020 and 2023 using data from the European Health Survey in Spain 2020 (EHSS 2020) and the Spanish National Health Survey 2023 (SNHS 2023). In addition, factors associated with vaccination in both periods were analyzed, and coverage rates were compared with those of a sample of individuals without diabetes matched by age, gender, and place of residence.

## 2. Materials and Methods

### 2.1. Study Design and Study Population

A descriptive cross-sectional study was conducted using data from the SNHS 2023. The SNHS 2023 is a nationwide survey aimed at a representative sample of the non-institutionalized civilian population aged 15 years or older, residing in main family dwellings throughout Spain. The survey employs a three-stage stratified cluster sampling design. In total, 21,032 individuals aged 15 years or older were selected, with a data collection period spanning from 1 August 2023, to 1 August 2024. The complete methodology of the SNHS 2023 is available on the website of the Ministry of Health [12].

To analyze temporal trends in influenza vaccination coverage among individuals with diabetes, data from the EHSS 2020 was also used. This survey follows a sampling design and data collection methodology equivalent to that of the SNHS 2023, allowing for comparisons between both periods. Detailed information on the methodology of the EHSS 2020 can be found on the Ministry of Health website [13].

All variables were collected through self-report, based on structured questions included in the standardized questionnaires of both surveys.

We considered participants to have diabetes if they answered “yes” to the question “Has a doctor ever told you that you have diabetes?”.

### 2.2. Matching Method

To control for potential confounding factors derived from differences in the distribution of age, gender, and region between individuals with and without diabetes, a one-to-one matching procedure was applied among participants from each survey. For each participant with diabetes included in the EHSS 2020 or the SNHS 2023, a subject without diabetes was selected who exactly matched the variables of gender, age, and autonomous community of residence. The process was carried out independently for each of the two surveys (2020 and 2023), thereby ensuring internal comparability between groups within each year. In cases where more than one eligible participant without diabetes was available for a participant with diabetes, the matched individual was selected randomly. This procedure allowed for the construction of two matched samples, ensuring homogeneity in the matching variables and improving the comparative validity of the analysis between both groups. Cases for which no exact match was found according to the defined criteria were excluded.

### 2.3. Study Variables

The variables analyzed in this study were based in identical questions in both surveys (EHSS 2020 and SNHS 2023), allowing for direct comparison. Influenza vaccination was considered the dependent variable and was defined based on an affirmative response to the question: “Were you vaccinated against influenza during the last vaccination campaign?”.

Independent variables were grouped into three blocks.

(1) Sociodemographic variables, namely: gender, age groups, marital status, educational level, and self-declared social status.
(2) Health-related variables: self-rated health status and body mass index (BMI), based on self-reported height and weight and obesity was considered when BMI ≥ 30 kg/m^2^.
(3) Presence of the following chronic diseases: myocardial infarction, respiratory diseases, cancer, and stroke (medical conditions were considered present when participants responded affirmatively to the question: “Has a doctor ever told you that you have or have had this disease?”).
(4) Lifestyle-related variables: alcohol consumption, smoking status, and leisure-time physical activity.

Detailed questions and categorical definitions for each study variable are described in Table S1.

### 2.4. Statistical Analysis

A descriptive analysis of the sociodemographic, clinical, and lifestyle-related characteristics of participants with and without diabetes was performed using absolute frequencies, percentages, and their corresponding 95% confidence intervals (95% CI).

To compare influenza vaccination coverage rates between groups with and without diabetes in each year (2020 and 2023), the Fisher exact test was used for categorical variables. Vaccination rates were also calculated according to subgroups defined by age, gender, and other study variables for both 2020 and 2023.

Subsequently, independent multivariable logistic regression models were constructed for the years 2020 and 2023 in order to identify factors associated with the probability of having received the influenza vaccine among the population with diabetes. In all models, the dependent variable was influenza vaccination (yes/no). Independent variables were selected based on their statistical significance in univariate analyses (*p* < 0.05) or their clinical relevance and support in the literature.

Additionally, to assess temporal changes in vaccination coverage between both years, a logistic regression model was built combining the matched samples from 2020 and 2023, including survey year as an independent variable, along with adjustment variables (gender, age, and other relevant factors). Crude and adjusted odds ratios (ORs) with their 95% CIs were calculated.

The steps described by Hosmer et al. [14] were followed to construct multivariable models. Model goodness of fit was evaluated using the Hosmer–Lemeshow test. Statistical analyses were performed using SPSS software version 26.0 (IBM Corp., Armonk, NY, USA). A *p* value < 0.05 was considered statistically significant in all analyses.

### 2.5. Ethical Considerations

In accordance with current Spanish legislation, approval or exemption from an ethics committee is not required for research using publicly available databases with freely accessible and anonymized information, as is the case for the EHSS 2020 and the SNHS 2023 [15]. Data protection and participant confidentiality were strictly ensured throughout the conduct of this study. Both datasets are provided by the Ministry of Health and are available for consultation and download through its institutional data portal (https://www.sanidad.gob.es/estadisticas/microdatos.do, accessed 6 January 2026).

## 3. Results

### 3.1. Characteristics of Participants with Diabetes in the SNHS 2023

Table 1 shows the distribution according to sociodemographic, health-related, and lifestyle-related characteristics for the 3774 matched pairs of participants with and without diabetes included in the SNHS 2023. The gender distribution was 52.7% women and 47.3% men, with most participants with diabetes being older than 60 years (80.6%). The population with diabetes exhibited a more unfavorable sociodemographic profile, with lower social status (low status: 52.1% vs. 48.2%) and lower educational level (primary education: 39.6% vs. 35.5%; secondary education: 46.7% vs. 46.4%; higher education: 13.8% vs. 18.2%).

Regarding health-related variables, the prevalence of obesity was significantly higher in the diabetes group (29.8% vs. 16.9%), as was the proportion of individuals who did not engage in leisure-time physical activity (44.9% vs. 37.1%). In addition, individuals with diabetes reported poorer self-rated health: only 39.3% rated their health as very good or good, compared with 56.7% in the group without diabetes, while 19.3% rated it as poor or very poor (vs. 11.9%). With respect to lifestyle habits, the diabetes group showed lower alcohol consumption (36.8% vs. 47.3%).

Furthermore, significantly higher prevalences of chronic comorbidities were observed among individuals with diabetes, including myocardial infarction, respiratory diseases, cancer, and stroke.

### 3.2. Trends in Influenza Vaccination Coverage Among the Spanish Population with Diabetes Between 2020 and 2023

Table 2 shows the evolution of influenza vaccination coverage in individuals with and without diabetes in Spain, based on data from the EHSS 2020 and SNHS 2023 and stratified by age group and gender.

The table reveals a significant increase in influenza vaccination coverage in 2023 compared with 2020 in both groups, with increases of 13.9 and 14.5 percentage points among those with and without diabetes, respectively. In both surveys, vaccination coverage was significantly higher among individuals with diabetes than in those without diabetes: 52.0% vs. 40.5% in 2020 and 65.9% vs. 55.0% in 2023.

When analyzed by age group, a progressive increase in coverage with age was observed across both surveys, reaching 74.7% among participants with diabetes aged 75 years or older. In all age ranges, vaccination coverage was higher in the diabetes population compared with non-diabetes matched subjects.

Regarding gender, a notable improvement in vaccination coverage was observed for both men and women with diabetes. In 2020, rates were 48.9% for men and 55.1% for women; in 2023, these figures increased to 64.4% and 67.3%, respectively. Differences compared with the non-diabetes groups were statistically significant for gender in both years.

### 3.3. Analysis of Influenza Vaccination Coverage Among Individuals with Diabetes in the SNHS 2023

Table 3 shows influenza vaccination coverage among individuals with and without diabetes in the National Health Survey of Spain 2023, according to sociodemographic variables, lifestyle, and health status.

As shown in Table 3, across all categories of the study variables, participants with diabetes had significantly higher vaccination coverage compared with subjects without diabetes, except among those who reported obesity, poor or very poor perceived health, or a diagnosis of myocardial infarction, respiratory diseases, cancer, or stroke.

Taken together, the results presented in Table 2 and Table 3 indicate that the categories with the highest influenza vaccination rates among the participants with diabetes in the SNHS 2023 were an age of 75 years or older, female gender, marital status “separated, divorced, or widowed,” primary education level, absence of alcohol consumption, no physical activity, self-rated health as “poor or very poor,” and having reported a diagnosis of myocardial infarction or stroke.

### 3.4. Multivariable Analysis of Influenza Vaccination Coverage in the Spanish Population with Diabetes

Table 4 presents the results of the multivariable logistic regression analysis used to identify predictors of adherence to influenza vaccination among individuals with diabetes, based on combined data from the EHSS 2020 and SNHS 2023.

Age was identified as a strong positive predictor of vaccination, with the strength of association increasing across age groups. An OR of 10.42 (95% CI: 6.71–16.18) was observed for individuals aged 75 years or older compared with the youngest group (18–44 years). The presence of comorbidities was also positively associated with vaccination, as a history of acute myocardial infarction and respiratory diseases were identified as positive predictors of influenza vaccination, with ORs of 1.43 (95% CI: 1.07–1.92) and 1.64 (95% CI: 1.30–2.07), respectively.

Smoking and alcohol consumption were identified as negative predictors of influenza vaccination, with lower likelihood of vaccination among smokers (OR: 0.70; 95% CI: 0.56–0.86) and alcohol consumers (OR: 0.84; 95% CI: 0.71–0.99).

Finally, the multivariable analysis demonstrated a significant improvement in influenza vaccine adherence between 2020 and 2023, confirming a significant increase in the probability of having received the vaccine in 2023 compared with 2020 (adjusted OR: 1.82; 95% CI: 1.56–2.12). In Table S2, the results of the initial logistic regression model are shown, including all the study variables that showed a significant association with influenza vaccination in participants with diabetes in the bivariate analysis (Table 2 and Table 3).

Table S3 shows the results of a multivariable logistic regression analysis identifying predictors of vaccination exclusively among participants with diabetes in the SNHS 2023. Results revealed that positive predictors for influenza vaccination were older age, with an OR of 17.73 (95% CI: 9.22–34.08) for persons with diabetes aged 75 years or older, and a history of myocardial infarction (OR: 2.08; 95% CI: 1.25–3.46).

## 4. Discussion

In the present study, influenza vaccination coverage among individuals with diabetes was analyzed using SNHS 2023 data, as well as temporal trends between 2020 and 2023 in Spain. Coverage among adults with diabetes reached 65.9% in 2023, representing an increase of 13.9% compared with 2020. This finding shows a notable improvement in vaccination coverage among the diabetes population; however, rates remain below the 75% recommended for at-risk populations [7,8]. This increase in vaccination rates may reflect a combination of factors, such as intensified vaccination campaigns, the inclusion of influenza vaccination within COVID-19 prevention strategies, and greater recommendation by healthcare professionals [16]. Additionally, after multivariable adjustment, we observed a nearly twofold increase in the probability of vaccination in 2023 compared with 2020 among individuals with diabetes. This finding suggests a widespread behavioral change across all age and gender groups, indicating that the COVID-19 pandemic may help to increase influenza vaccine adherence in the population with diabetes [17,18]. De la Cámara et al. recently described a similar change in influenza vaccination trends in Spain during the COVID-19 pandemic, suggesting increased awareness or risk perception during this period [19]. Consistent with the present study, a generalized increase in influenza vaccination coverage has also been observed across Europe following the onset of the COVID-19 pandemic, particularly in at-risk populations [17,20,21]. For example, in France, coverage increased from 52.0% in 2019 to 59.9% in 2021 [22], while in Germany, among individuals over 60 years, coverage rose from approximately 40% in 2019/20 to 43.3% in 2021/22 [23]. In Italy, among healthcare professionals, coverage increased from 12.8% in 2019/20 to 40.9% in 2020/21 [24].

Individuals with diabetes in Spain have shown significantly higher influenza vaccination coverage compared with the non-diabetes population in our study. In 2020, 52.0% of individuals with diabetes were vaccinated versus 40.5% of their non-diabetes counterparts, and these figures increased to 65.9% and 55.0%, respectively, in 2023. This pattern aligns with findings from previous studies [6,25].

Higher vaccination adherence in populations with elevated risk profiles supports the notion that perceived disease severity and the prioritization established by health policies are critical factors influencing vaccine acceptance [26].

In this study, various predictors of adherence to influenza vaccination were described. Age was identified as the main positive predictor, with adherence increasing in correlation with older age among individuals with diabetes. After multivariable adjustment, the probability of vaccination among subjects with diabetes aged 65–74 years was more than five times higher compared with the 18–44-year age group, and more than ten times higher in patients with diabetes aged 75 years or older.

The increase in influenza vaccination coverage with advancing age has been consistently observed in Spain, both in the general population and among individuals with high-risk conditions [6,19,25,27,28,29,30]. Previous studies suggest that this pattern may be explained by greater contact with healthcare services as age increases, thereby enhancing opportunities for healthcare professionals to recommend vaccination. In addition, awareness of influenza severity and vaccine effectiveness tends to improve with age, partly due to sustained public health education campaigns. Finally, older individuals often exhibit a higher perceived susceptibility to infection [27,31,32,33,34]. In Spain, influenza vaccination coverage among individuals aged ≥60 years is reinforced each autumn through coordinated strategies implemented at both population and individual levels by national and regional health authorities and primary care services. Population-level interventions include mass media campaigns (radio, television, and digital platforms), as well as informational materials distributed in healthcare and social care settings. At the individual level, strategies involve telephone calls, text message reminders, active recommendations during appointment scheduling, and opportunistic vaccination during routine healthcare visits. Additional measures include electronic health record alerts and e-prescribing prompts, expansion of vaccination sites and opening hours, deployment of mobile vaccination units, and provision of home-based vaccination for individuals with limited mobility [6,19,25,27,28,29,30].

Diagnoses of acute myocardial infarction and respiratory diseases were also identified as positive predictors of influenza vaccination among the diabetes population. It is important to note that both conditions are closely associated with age. However, the direct association of respiratory diseases with a higher risk of influenza-related complications—and the consequent prioritization of vaccination for this high-risk group—may explain the observed relationship [35,36].

Regarding behavioral habits, consistent with previous findings, the present study identified that smoking and alcohol consumption were associated with a lower likelihood of vaccination. This suggests that both individuals with and without diabetes with unhealthy habits exhibit lower health risk perception and tend to show reduced adherence to vaccination recommendations [37,38,39].

The results underscore the need to maintain and strengthen vaccination strategies among the diabetes population, with particular focus on less adherent subgroups: individuals under 60 years of age, those with unhealthy lifestyles, or without comorbidities. More effective health education and improved accessibility and follow-up in primary care are required, as medical advice is one of the most significant influences on vaccination decisions [40,41,42,43,44]. Strategies such as postal reminders, telephone calls, emails, home visits, and educational interventions about influenza risks and the need for immunization have proven effective [6]. In addition, digital health tools, such as mobile applications or patient portals, offer novel and efficient solutions to support preventive education [45,46,47].

The main strengths of this study are its large, nationally representative sample and the inclusion of sociodemographic and lifestyle variables, some of which are not routinely collected in health records. However, several limitations should be noted. Data from the EHSS and SNHS may be subject to non-response, recall, or social desirability biases; information on reasons for non-vaccination is not available, limiting the development of targeted strategies; and there is no formal validation of the questions used to classify diabetes, although self-report has shown good sensitivity and specificity [48]. Furthermore, cross-sectional design hinders causal inferences, and reverse causality should be considered. Despite these well-known limitations of health surveys, these databases represent a valid source of information for assessing adherence to influenza vaccination programs among the diabetes population, as well as for identifying predictors of influenza vaccination. Researchers from different countries have periodically used health surveys to evaluate influenza vaccination coverage [6,30,49].

## 5. Conclusions

Influenza vaccination coverage among the Spanish population with diabetes increased significantly between 2020 and 2023, in parallel with behavioral and healthcare changes resulting from the COVID-19 pandemic. Nevertheless, coverage rates remain below international targets, highlighting the need for more personalized and sustained strategies to promote vaccination. Policymakers should maintain broad population-based vaccination strategies while prioritizing efforts to reduce inequities among individuals with diabetes. Targeted interventions should focus on younger patients and those with unhealthy lifestyles, who are consistently less likely to be vaccinated. It is essential to improve health education for both patients and healthcare professionals, enhance accessibility to healthcare services, and strengthen proactive vaccine recommendations by providers. In addition, the use of reminder systems—including postal letters, postcards, and digital tools—should be expanded to increase adherence and move toward optimal vaccination coverage in this high-risk population.

## Figures and Tables

**Table 1:** Distribution of study population according to study variables in subjects residing in Spain with diabetes and matched participants without diabetes included in the Spanish National Health Survey 2023 (SNHS 2023).

Variables	Categories	Diabetes	No Diabetes	TOTAL	*p* Value
		N (%)	N (%)	N (%)	
Age groups (years)	18–44	90 (4.8)	90 (4.8)	180 (4.8)	NA
	45–59	277 (14.6)	277 (14.6)	552 (14.6)	
	60–74	762 (40.4)	762 (40.4)	1522 (40.4)	
	75 or older	758 (40.2)	758 (40.2)	1514 (40.2)	
Gender	Male	893 (47.3)	893 (47.3)	1786 (47.3)	NA
	Female	994 (52.7)	994 (52.7)	1988 (52.7)	
Social status	Upper	233 (12.9)	324 (18.1)	557 (15.5)	<0.001
	Medium	631 (35.0)	603 (33.7)	1234 (34.4)	
	Lower	939 (52.1)	861 (48.2)	1800 (50.1)	
Marital status	Single	253 (13.4)	279 (14.8)	532 (14.1)	0.130
	Married	935 (49.5)	965 (51.1)	1900 (50.3)	
	Other *	699 (37.0)	643 (34.1)	1342 (35.6)	
Educational level	Primary	616 (39.6)	572 (35.5)	1188 (37.5)	0.001
	Secondary	726 (46.7)	748 (46.4)	1474 (46.5)	
	Higher	214 (13.8)	293 (18.2)	507 (16.0)	
Obesity	No	1246 (70.2)	1483 (83.1)	2729 (76.7)	<0.001
	Yes	529 (29.8)	302 (16.9)	831 (23.3)	
Physical activity	No	838 (44.9)	691 (37.1)	1529 (41.0)	<0.001
	Yes	1028 (55.1)	1171 (62.9)	2199 (59.0)	
Smoking habits	No	1642 (87.2)	1624 (86.6)	3266 (86.9)	0.536
	Yes	240 (12.8)	252 (13.4)	492 (13.1)	
Alcohol consumption	No	1184 (63.2)	983 (52.7)	2167 (58.0)	<0.001
	Yes	688 (36.8)	884 (47.3)	1572 (42.0)	
Self-rated health	Very good or good	742 (39.3)	1069 (56.7)	1811 (48.0)	<0.001
	Regular	780 (41.3)	593 (31.4)	1373 (36.4)	
	Poor or very poor	365 (19.3)	225 (11.9)	590 (15.6)	
Myocardial infarction	No	1730 (91.7)	1825 (96.7)	3555 (94.2)	<0.001
	Yes	157 (8.3)	62 (3.3)	219 (5.8)	
Respiratory diseases	No	1629 (86.4)	1725 (91.5)	3354 (88.9)	<0.001
	Yes	257 (13.6)	161 (8.5)	418 (11.1)	
Cancer	No	1698 (90.0)	1748 (92.6)	3446 (91.3)	0.004
	Yes	189 (10.0)	139 (7.4)	328 (8.7)	
Stroke	No	1784 (94.5)	1814 (96.1)	3598 (95.3)	0.021
	Yes	103 (5.5)	73 (3.9)	176 (4.7)	

* Other: widower or separated or divorced. NA: not applicable. Fisher exact test was used to compare proportions. *p* values represent the difference between participants with diabetes and matched without this condition.

**Table 2:** Influenza vaccination coverage across European Health Survey 2020 (EHS 2020) and Spanish National Health Survey (SNHS 2023) according to age and gender among participants with and without diabetes.

Variables	Categories	EHS 2020		*p* Value	SNHS 2023		*p* Value	TOTAL		*p* Value
		Diabetes	No Diabetes		Diabetes	No Diabetes		Diabetes	No Diabetes	
		N (%)	N (%)		N (%)	N (%)		N (%)	N (%)	
Age groups (years)	18–44	17 (20.7)	8 (9.8)	0.081	16 (17.8)	10 (11.1)	0.289	33 (19.2)	18 (10.5)	0.033
	45–59	99 (29.6)	27 (8.1)	<0.001	97 (35.1)	50 (18.1)	<0.001	196 (32.1)	77 (12.6)	<0.001
	60–74	432 (50.3)	313 (36.4)	<0.001	506 (66.5)	403 (53.0)	<0.001	938 (57.9)	716 (44.2)	<0.001
	75 or older	519 (67.1)	484 (62.5)	0.070	624 (82.4)	575 (76.0)	0.002	1143 (74.7)	1059 (69.2)	<0.001
Gender	Male	499 (48.9)	401 (39.3)	<0.001	575 (64.4)	447 (50.1)	<0.001	1074 (56.1)	848 (44.3)	<0.001
	Female	569 (55.1)	431 (41.7)	<0.001	669 (67.3)	591 (59.5)	<0.001	1238 (61.1)	1022 (50.4)	<0.001
Total per survey		1068 (52.0)	832 (40.5)	<0.001	1244 (65.9)	1038 (55.0)	<0.001	2312 (58.7)	1870 (47.5)	<0.001

Fisher exact test was used to compare proportions. *p* values represent the difference between participants with diabetes and matched participants without this condition.

**Table 3:** Influenza vaccination coverage in the Spanish National Health Survey 2023 (SNHS 2023) according to sociodemographic and health status variables among participants with and without diabetes.

Variable	Category	Diabetes		No Diabetes		*p* Value
		N (%)	95% CI	N (%)	95% CI	
Marital status ^a,b^	Single	142 (56.1)	50.0–62.1	107 (38.4)	32.8–44.2	<0.001
	Married	610 (65.2)	62.1–68.2	531 (55.0)	51.9–58.1	<0.001
	Other *	492 (70.4)	66.9–73.7	400 (62.2)	58.4–65.9	0.002
Educational level ^a,b^	Primary	455 (73.9)	70.3–77.2	363 (63.5)	59.5–67.3	<0.001
	Secondary	392 (54.0)	50.4–57.6	326 (43.6)	40.1–47.2	<0.001
	Higher	136 (63.6)	57.0–69.8	146 (49.8)	44.1–55.5	0.003
Social status	Upper	145 (62.2)	55.9–68.3	165 (50.9)	45.5–56.3	0.009
	Medium	428 (67.8)	64.1–71.4	316 (52.4)	48.4–56.4	<0.001
	Lower	606 (64.5)	61.4–67.5	495 (57.5)	54.2–60.8	0.002
Obesity ^a,b^	No	848 (68.1)	65.4–70.6	793 (53.5)	50.9–56.0	<0.001
	Yes	326 (61.6)	57.4–65.7	187 (61.9)	56.4–67.3	0.941
Smoking habits ^a,b^	No	570 (68.0)	64.8–71.1	399 (57.7)	54.0–61.4	<0.001
	Yes	663 (64.5)	61.5–67.4	629 (53.7)	50.9–56.6	<0.001
Alcohol consumption ^a,b^	No	1129 (68.8)	66.5–71.0	955 (58.8)	56.4–61.2	<0.001
	Yes	113 (47.1)	40.8–53.4	80 (31.7)	26.2–37.7	<0.001
Physical activity	No	828 (69.9)	67.3–72.5	617 (62.8)	59.7–65.7	<0.001
	Yes	409 (59.4)	55.7–63.1	418 (47.3)	44.0–50.6	<0.001
Self-rated health ^a,b^	Very good or good	447 (60.2)	56.7–63.7	516 (48.3)	45.3–51.3	<0.001
	Regular	532 (68.2)	64.9–71.4	369 (62.2)	58.3–66.1	<0.022
	Poor or very poor	265 (72.6)	67.9–77.0	153 (68.0)	61.7–73.8	0.263
Myocardial infarction ^a,b^	No	1121 (64.8)	62.5–67.0	994 (54.5)	52.2–56.7	<0.001
	Yes	123 (78.3)	71.4–84.2	44 (71.0)	58.9–81.1	0.291
Respiratory diseases ^a,b^	No	1060 (65.1)	62.7–67.4	933 (54.1)	51.7–56.4	<0.001
	Yes	184 (71.6)	65.9–76.8	104 (64.6)	57.0–71.7	0.158
Cancer ^b^	No	1107 (65.2)	62.9–67.4	942 (53.9)	51.5–56.2	<0.001
	Yes	137 (72.5)	65.8–78.5	96 (69.1)	61.0–76.3	0.539
Stroke ^a,b^	No	1164 (65.2)	63.0–67.4	986 (54.4)	52.1–56.6	<0.001
	Yes	80 (77.7)	68.9–84.9	52 (71.2)	60.2–80.6	0.379

^a^ Significant association for vaccination coverage in population diagnosed with diabetes; ^b^ significant association for vaccination coverage in population without diabetes mellitus; * other: widower or separated or divorced. CI: confidence interval. Fisher exact test was used to compare proportions.

**Table 4:** Multivariable analysis for variables associated with vaccination adherence and time trends among participants with diabetes included in the EHS2020 and SNHS 2023.

Variables	Categories	OR (95% CI)
Gender	Male	1
	Female	1.01 (0.85–1.18)
Age groups (years)	18–44	1
	45–59	2.09 (1.35–3.23)
	60–74	5.67 (3.73–8.62)
	75 or older	10.42 (6.71–16.18)
Smoking habits	No	1
	Yes	0.70 (0.56–0.86)
Alcohol consumption	No	1
	Yes	0.84 (0.71–0.99)
Myocardial infarction	No	1
	Yes	1.43 (1.07–1.92)
Respiratory diseases	No	1
	Yes	1.64 (1.30–2.07)
Survey	EHS 2020	1
	SNHS 2023	1.82 (1.56–2.12)

Other: widower or separated or divorced. EHS: European Health Survey; SNHS: Spanish National Health Survey; CI: Confidence interval; OR: Odd ratio.

## Data Availability

Data of the European Health Survey in Spain 2020 and the Spanish National Health Survey 2023 can be freely downloaded from https://www.sanidad.gob.es/estadisticas/microdatos.do (accessed 22 December 2025).

## Supplementary Materials

The following supporting information can be downloaded at https://www.mdpi.com/article/10.3390/epidemiologia7020053/s1: Table S1: Definition of study variables used in our investigation according to the questions included in the European Health Survey for Spain 2020 and the Spanish National Health Interview Survey for year 2023; Table S2: Initial multivariable logistic regression model to identify predictors of vaccination adherence for participants with diabetes included in the SNHS 2020 and 2023; Table S3: Multivariable analysis for vaccination adherence for participants with diabetes included in the SNHS 2023.

## Abbreviations

The following abbreviations are used in this manuscript:

EHSS 2020	European Health Survey in Spain 2020
SNHS 2023	Spanish National Health Survey 2023
BMI	Body mass index
