# Supplementary material for: What Drives Influenza Vaccination in People with Diabetes? Evidence from the National Health Surveys of 2020 and 2023 in Spain
Source: Epidemiologia (Basel). 2026 Apr 8;7(2):53. doi: 10.3390/epidemiologia7020053 (PMC13114704; doi:10.3390/epidemiologia7020053)
Supplement: Supplementary file 1 [file epidemiologia-07-00053-s001.zip › epidemiologia-4206586-supplementary.pdf]

**Table S1.** Definition of study variables used in our investigation according to the questions included in the European Health Survey for Spain 2020 and the Spanish National Health Interview Survey for year 2023.

| QUESTIONS | DESCRIPTION AND ANSWER | VARIABLE NAME | CATEGORIES |
|---|---|---|---|
| Were you vaccinated against influenza during the last vaccination campaign? | Those that answered "Yes" were considered vaccinated. | Influenza vaccination | No<br>Yes |
| Which is your sex? | Man<br>Woman | Gender | Male: when answered "man".<br>Female: when answered "woman". |
| How old are you? | Age in years | Age groups | 18-54<br>55-64<br>65-74<br>75 or older |
| What is your legal civil status? | 1. Single<br>2. Married<br>3. Widower<br>4. Separated<br>5. Divorced | Marital status | Single: option 1<br>Married: option 2<br>Other: option 3,4 and 5. |
| Please, indicate the highest level of education you have completed. | 1. Does not know how to read or write<br>2. Incomplete primary education<br>3. Complete primary education<br>4. First stage of Secondary Education, with or without a qualification<br>5. Elementary Spanish Upper Secondary Education<br>6. Upper secondary education<br>7. Intermediate vocational training or equivalent<br>8. Advanced vocational training or equivalent<br>9. University studies or equivalent<br>10. Over university (master, PhD….) | Educational level | 1. Primary school or less: Options 1 to 3<br>2. Secondary school or equivalent: Options 4 to 8<br>3. Higher education: Options 9 and 10 |
| What occupation, profession or job do you perform in your actual position? | Occupations are coded following the National Classification of Occupations 2011 (CNO2011) | Social status | 1. Upper<br>2. Medium<br>3. Lower |
| #1. Could you tell me what your height is, without shoes?<br>#2. Could you tell me how much you weigh, without clothes or shoes? | Body mass index is calculated with height and weight | Obesity | 1. Yes: If body mass index is 30 or over<br>2. No: If body mass index is under 30 |
| Could you tell me if you smoke? | 1. Yes, I smoke daily<br>2. Yes, I smoke, but not daily<br>3. I don't currently smoke but have smoked before | Smoking habits | 1. Yes: Options 1 and 2<br>2. No: Options 3 and 4 |

| | | | |
|---|---|---|---|
| | 4. I neither smoke nor have I ever smoked regularly | | |
| During the past 12 months, how often have you had alcoholic beverages of any kind (i.e. beer, wine, spirits, distilled and mixed drinks, or other alcoholic beverages)? | 1. Daily or almost daily<br>2. 5-6 days per week<br>3. 3-4 days per week<br>4. 1-2 days per week<br>5. 2-3 days in a month<br>6. Once a month<br>7. Less than once a month<br>8. Not in the last 12 months, have I stopped drinking<br>9. Never or just a few sips to taste it throughout life | Alcohol consumption | 1. Yes: Options 1 to 6<br>2. No: Option 7 to 9 |
| Which of these possibilities best describes how often you do some physical activity in your free time? | 1. I don't exercise. I occupy my free time almost completely sedentary<br>2. I do some occasional physical or sports activity<br>3. I do physical activity several times a month<br>4. I do sports or physical training several times a week | Physical activity | 1. No: Option 1<br>2. Yes: Option 2 to 4 |
| In the last 12 months, how would you rate your health? | 1. Very good<br>2. Good<br>3. Fair<br>4. Bad<br>5. Very bad | Self-rated Health | 1. Very good/good: Options 1 and 2<br>2. Fair/poor/very poor: Options 3 to 5<br>1. Very good/good: Options 1 and 2 |
| #1.Do you have, or have you ever had any of the following diseases or medical conditions?<br>Those who answered “yes” completed question #2<br><br>#2.Have you suffered from that disease/medical condition over the last 12 months?<br>Those who answered “yes” completed question #3<br><br>#3. Have you been diagnosed by a physician with this disease/health condition? | A list of 32 conditions was given to the interviewee including: myocardial infarction | Myocardial infarction | Yes: when answered affirmatively to the question #3 for myocardial infarction.<br>No: any other given answer |
| | A list of 32 conditions was given to the interviewee including: asthma, emphysema, chronic obstructive pulmonary disease and/or chronic bronchitis. | Respiratory diseases | Yes: when answered affirmatively to the question for any of the listed chronic conditions.<br><br>No: any other given answer. |
| | A list of 32 conditions was given to the interviewee including Diabetes Mellitus. | Diabetes Mellitus | Yes: when answered affirmatively to the question #3 for diabetes mellitus.<br>No: any other given answer |
| | A list of 32 conditions was given to the interviewee including: Malignant Tumors | Cancer | Yes: when answered affirmatively to the question for Malignant Tumors.<br>No: any other given answer. |
| | A list of 32 conditions was given to the interviewee including: stroke | Stroke | Yes: when answered affirmatively to the question for Stroke.<br><br>No: any other given answer |

**Table S2.** Initial multivariable logistic regression model to identify predictors of vaccination adherence for participants with diabetes included in the SNHS 2020 and 2023

| VARIABLE | CATEGORY | OR 95% CI |
|---|---|---|
| Gender | Male | 1 |
| | Female | 1.01 (0.85-1.20) |
| Age groups (years) | 18-44 | 1 |
| | 45-59 | 2.31 (1.48-3.61) |
| | 60-74 | 6.15 (4.01-9.45) |
| | 75 or older | 11.44 (7.29-17.94) |
| Marital status | Single | 1 |
| | Married | 1.13 (0.89-1.43) |
| | Other* | 0.90 (0.69-1.17) |
| Educational level | Primary | 1 |
| | Secondary | 1.15 (0.67-1.44) |
| | Higher | 1.01 (0.76-1.30) |
| Obesity | No | 1 |
| | Yes | 0.97 (0.82-1.14) |
| Smoking habits | No | 1 |
| | Yes | 0.66 (0.54-0.82) |
| Alcohol consumption | No | 1 |
| | Yes | 0.83 (0.71-0.99) |
| Self-Rated Health | Very good or good | 1 |
| | Regular | 0.85 (0.69-1.06) |
| | Poor or very poor | 0.92 (0.74-1.14) |
| Myocardial infarction | No | 1 |
| | Yes | 1.57 (1.16-2.12) |
| Respiratory diseases | No | 1 |
| | Yes | 1.66 (1.31-2.09) |
| Stroke | No | 1 |
| | Yes | 1.38 (0.97-1.96) |
| Survey | EHS 2020 | 1 |
| | SNHS 2023 | 1.89 (1.62-2.20) |

The variables included in the initial model were those significantly associated with influenza vaccination in the bivariate analysis (Tables 2 and 3)

Table S3. Multivariable analysis for vaccination adherence for participants with diabetes included in the SNHS 2023

| VARIABLES | CATEGORIES | OR (95% IC) |
|---|---|---|
| Gender | Male | 1 |
| | Female | 0.97 (0.74-1.27) |
| Age groups (years) | 18-44 | 1 |
| | 45-59 | 2.81 (1.48–5.33) |
| | 60-74 | 8.79 (4.74–16.31) |
| | 75 or older | 17.73 (9.22–34.08) |
| Myocardial infarction | No | 1 |
| | Yes | 2.08 (1.25–3.46) |

SNHS: Spanish National Health Survey; NS: No significant; CI: Confidence interval; OR: Odd ratio.
